# Individual and neighborhood risk factors of hospital admission and death during the COVID-19 pandemic: a population-based cohort study

**DOI:** 10.1186/s12916-022-02715-4

**Published:** 2023-01-04

**Authors:** Max Bell, Maria-Pia Hergens, Stefan Fors, Per Tynelius, Antonio Ponce de Leon, Anton Lager

**Affiliations:** 1 Department of Perioperative Medicine and Intensive Care, Karolinska University Hospital, Stockholm, Sweden; 2 Department of Physiology and Pharmacology, Karolinska Institutet, Stockholm, Sweden; 3 Department of Global Public Health, Karolinska Institutet, Stockholm, Sweden; 4 Department of Communicable Disease Control and Prevention, Region Stockholm, Stockholm, Sweden; 5 Aging Research Center, Karolinska Institutet & Stockholm Universitet, Solna, Sweden; 6 Center for Epidemiology and Community Medicine, Region Stockholm, Stockholm, Sweden

**Keywords:** COVID-19, Socioeconomics, Population study, Cohort study, Stockholm, Epidemiology, Hospitalization, Mortality

## Abstract

**Background:**

The coronavirus disease 2019 (COVID-19) disproportionately affects minority populations in the USA. Sweden — like other Nordic countries — have less income and wealth inequality but lacks data on the socioeconomic impact on the risk of adverse outcomes due to COVID-19.

**Methods:**

This population-wide study from March 2020 to March 2022 included all adults in Stockholm, except those in nursing homes or receiving in-home care. Data sources include hospitals, primary care (individual diagnoses), the Swedish National Tax Agency (death dates), the Total Population Register “RTB” (sex, age, birth country), the Household Register (size of household), the Integrated Database For Labor Market Research “LISA” (educational level, income, and occupation), and SmiNet (COVID data). Individual exposures include education, income, type of work and ability to work from home, living area and living conditions as well as the individual country of origin and co-morbidities. Additionally, we have data on the risks associated with living areas. We used a Cox proportional hazards model and logistic regression to estimate associations. Area-level covariates were used in a principal component analysis to generate a measurement of neighborhood deprivation. As outcomes, we used hospitalization and death due to COVID-19.

**Results:**

Among the 1,782,125 persons, male sex, comorbidities, higher age, and not being born in Sweden increase the risk of hospitalization and death. So does lower education and lower income, the lowest incomes doubled the risk of death from COVID-19. Area estimates, where the model includes individual risks, show that high population density and a high percentage of foreign-born inhabitants increased the risk of hospitalization.

**Conclusions:**

Segregation and deprivation are public health issues elucidated by COVID-19. Neighborhood deprivation, prevalent in Stockholm, adds to individual risks and is associated with hospitalization and death. This finding is paramount for governments, agencies, and healthcare institutions interested in targeted interventions.

**Supplementary Information:**

The online version contains supplementary material available at 10.1186/s12916-022-02715-4.

## Background

Globally, it is known that countries with high levels of income inequality have performed significantly worse when dealing with the coronavirus disease 2019 (COVID-19) outbreak in terms of cases and deaths [1, 2]. Moreover, severe acute respiratory syndrome coronavirus 2 (SARS-CoV-2), causing COVID-19 disproportionately affects minority populations in the USA and in the UK [3–5].

During the 2009 H1N1, influenza reports in the USA of racial ethnic disparities with regard to complications and hospitalization [6] led to a national survey documenting the sources of these inequalities [7]. Increased risk of exposure to the virus and increased susceptibility to severe consequences of the infection combined with lack of health care access were important contributors.

In the rest of Western Europe and specifically in the Nordics, there is a paucity of data regarding the socioeconomic impact on the risk of infection, hospitalization, and mortality due to COVID-19. Even though the risk of COVID-19 varies enormously between geographical areas as well as between individuals with different sociodemographic characteristics, such as sex, age, and socioeconomic status, the relationship between geographical areas and individual risk factors is unknown.

The present study sought to address this knowledge gap with a population-wide study of all adult inhabitants in the Stockholm Region, excluding older adults living in nursing homes or receiving in-home care. We used the real-time COVID-19 monitoring framework in Region Stockholm to investigate the demographic and socioeconomic impact on the risk of hospitalization and mortality.

We hypothesized that demographic and socioeconomic factors contributed to the risk of hospitalization and death independently of other co-morbidity-related risk factors. We further wanted to assess if living areas carried additional risks of COVID hospitalization or death, even when adjusting for all individual factors.

## Methods

### Data sources

Stockholm’s administrative organization (Region Stockholm), responsible for all healthcare within the region, manages VAL (Swedish: Vård Analys Lager, the Stockholm Regional Healthcare Data Warehouse), a data warehouse of healthcare utilization. VAL contains complete hospital inpatient and hospital outpatient data, and primary care information, including consultations and diagnoses at the individual level. The coverage in VAL for inpatient care is over 99% [8] and the validity of the diagnostic coding is 85–95%, depending on the diagnosis [9]. The VAL database includes linkage to the Swedish National Tax Agency (death dates) and was also further linked to national population registers held by Statistics Sweden. This included the Total Population Register “RTB” (sex, age, birth country) [10]. RTB also contains Household Register data on geographical information, including street address and apartment data (size of household) and the Integrated Database For Labor Market Research ”LISA” (educational level, income, and occupation) [11]. In addition, we used data from SmiNet which is a national electronic surveillance system for reporting communicable diseases [12]. Since February 1, 2020, it is mandatory for the Swedish laboratories to report all PCR-confirmed cases of COVID-19 to SmiNet. All register linkage used the unique personal identity number given to each Swedish citizen [13].

### Study population

The study population consisted of all individuals 18 years and older, residing in Stockholm County during a calendar year from March 1st, 2020, to February 28th, 2021, based on data from the Population Register [10]. Individuals permanently staying in nursing homes were excluded since they were mainly treated for COVID-19 in their facilities and hence did not contribute risk for hospitalization. Also, those with home-care services were excluded due to increased risk of infection, uncoupled to sociodemographic status.

### Variables used as exposure and confounders in statistical models

Educational level: separated into low (pre-secondary education), medium (secondary-), and higher education (post-secondary education) based on LISA data.

Income: household disposable income (LISA data) was separated into quintiles, from the 20% with the lowest income to the 20% with the highest income defined as income including welfare, after taxation.

Work: using the “standard for Swedish occupational classifications” (SSYK), based on the “international standard classification of occupations” (ISCO), we dichotomized individuals based on the ability to work from home or not. Additionally, we have analyzed healthcare workers, and adults not working (full-time students, unemployed, on long-term sick leave, or retired) separately. These classifications were made by individually assessing the work characteristics of the different occupations.

Living area: the greater Stockholm area was divided into 164 neighborhoods with an average of 14,000 inhabitants. They were ranked after death due to COVID-19 per 10,000 people (excluding those living at nursing homes) and then grouped into quintiles. In other analyses, we introduce them separately, as baseline hazards.

Living condition: measured as the size of household  (the number of people in the household).

Country of origin: data on country and region of origin is available, but for these analyses, we divided subjects into those born in Sweden and not born in Sweden.

Co-morbidities: the following ICD codes from the VAL database were chosen, based on previous risk factor publications [14, 15] heart failure (I50), ischemic heart disease (I20-25), diabetes (E10-14), obesity (E66), chronic kidney disease (N18), chronic obstructive pulmonary disease (J44), cancer (C00-97) and liver disease (I85–I85.9; I98.2; K79–K71, K71.3–K77.8; R16–R18.9; Z52.6; Z94.4). All individuals with one of these diagnoses within the last 5 years (or 2 years for cancer) or one hospitalization due to cardiovascular disease have been classified as having a co-morbidity.

### Outcomes

The primary outcome was 30-day all-cause mortality after laboratory-confirmed COVID-19 infection. The secondary outcome was hospitalization with confirmed COVID-19 infection. Hospitalization as outcome was verified via SmiNet and validated if inpatient treatment included the emergency ICD10 codes for COVID-19 (issued by the WHO): U07.1–U07.2 as the main diagnosis.

### Observation period

The observation period ran from the 1st of March 2020 to the 28th of February 2021. Follow-up ended at loss to follow-up (emigration from the Stockholm Region), end of study, or the date of outcomes.

### Statistical analyses

Multivariate logistic regression model was used, fitted for each outcome. The modeling strategy consisted of analyzing a selection of individual covariates first, followed by the same individual covariates and a selection of area-level covariates. To avoid collinearity in the latter part of the modeling, each area-level covariate was included one at a time, always including the whole set of individual covariates. Area-level covariates outside of area percentages of children, percentages of elderly, density, and inhabitants born outside of Sweden were included in a principal component analysis to generate a composite neighborhood deprivation score (NDS), divided into three levels, from least (NDS 1) to most deprived (NDS 3).

In Additional file 1: Table S1 associations were estimated using a Cox proportional hazards model with income as exposure and adjusted for confounder variables as categorical variables and stratified for area effects, thus allowing for different baseline hazards in each area. In this analysis, individuals were censored at the date of death from other causes, emigration from the region, or at the end of follow-up, whichever came first. The potential confounders were added sequentially in order to show the confounding impact of different domains of the sociodemographic factors.

### Ethical approval

The study has been approved by the Regional Ethical Review Board, Stockholm (2021–00810). All data were analyzed in a pseudonymized format and confidentiality was always maintained. Reporting follows the STrengthening the Reporting of OBservational studies in Epidemiology and the REporting of studies Conducted using Observational Routinely-collected health Data statements [16, 17].

### Data sharing

Swedish privacy law prohibits us from making registered data publicly available.

## Results

In all, 1.7 million people were followed from March 1st, 2020, to February 28th, 2021, during which time 10,495 hospitalizations and 1148 deaths due to COVID-19 were registered. Tables 1 and 2 detail the *individual effect estimates* on covid-19 hospital admission and mortality. Men had a higher risk of both hospitalization and death than women, with relative risk (RR) 1.6 (confidence interval (CI) 1.53–1.66) and RR 2.5 (CI 2.19–2.85), respectively. Increasing age was strongly associated with increasing risks, mainly regarding to mortality where a relative risk of 53 was seen in the ≥75 years age category. Immigrants to Sweden were generally at higher risks of both outcomes, with a RR for hospitalization of 2.11 (2.02–2.21) and RR for death was 1.68 (1.47–1.92). Lower education and lower income levels were significantly coupled with higher relative risks of mortality. The lowest income level was associated with a doubled risk of death from COVID-19, and the presence of co-morbidities more than quadrupled the mortality risk.

**Table 1:** Frequency table for COVID-19 hospitalizations and mortality among a Stockholm Regions cohort of 1,782,125 persons followed between the 1st of March 2020 to the 28th of February 2021

	*N* (%)	Number of hospital admissions during follow-up (%)	Number of deaths during follow-up (%)
Total	1,782,125	10,495	1148
**Sex**			
Women	890,223 (50%)	4145 (39%)	351 (31%)
Men	891,902 (50%)	6350 (61%)	797 (69%)
**Age groups**			
18–44 years	878,906 (49%)	2021 (19%)	31 (3%)
45–59 years	459,285 (26%)	3065 (29%)	110 (10%)
60–74 years	311,575 (17%)	3239 (31%)	335 (29%)
75–110 years	132,359 (7%)	2170 (21%)	672 (59%)
**Region of origin (birth)**			
Sweden	1,226.715 (69%)	5225 (50%)	658 (57%)
Not born in Sweden	555,410 (31%)	5270 (50%)	490 (43%)
**Living conditions**^a^			
Single household	367,841 (21%)	2276 (22%)	385 (34%)
Double	524,238 (29%)	3483 (33%)	496 (43%)
Triple	301,768 (17%)	1538 (15%)	113 (10%)
Quartet	315,376 (18%)	1382 (13%)	54 (5%)
Multiple	272,902 (15%)	1816 (17%)	100 (9%)
**Education**			
High	689,193 (39%)	2957 (28%)	252 (22%)
Medium	761,873 (43%)	4505 (43%)	468 (41%)
Low	226,960 (13%)	2267 (22%)	329 (29%)
Other	56,279 (3%)	411 (4%)	71 (6%)
NA	47,820 (3%)	355 (3%)	28 (2%)
**Type of work**^b^			
Full-time home	510,967 (29%)	2725 (26%)	114 (10%)
Part-time home	475,712 (27%)	1416 (13%)	62 (5%)
Health worker	117,788 (17%)	837 (8%)	26 (2%)
Other^b^	677,658 (38%)	5520 (53%)	946 (82%)
**Income**			
Income Q5 (highest income)	347,377 (19%)	1528 (15%)	116 (10%)
Income Q4	346,117 (19%)	1531 (15%)	114 (10%)
Income Q3	340,517 (19%)	1832 (17%)	174 (15%)
Income Q2	323,936 (18%)	2382 (23%)	359 (31%)
Income Q1	326,115 (18%)	2698 (26%)	351 (31%)
No info on income	98,063 (6%)	524 (5%)	34 (3%)
**Comorbidities**			
No	1,447,390 (81%)	4933 (47%)	239 (21%)
Yes	334,735 (19%)	5562 (53%)	909 (79%)

^a^Living conditions are measured as the size of the household (i.e., the number of people in the household)

^b^Possibility to work from home full-time or part-time. ‘Other’ includes full-time students, unemployed, on long-term sick leave, or retired

**Table 2:** Relative risks^1^ with 95% confidence intervals for COVID-19 hospitalizations and mortality among a Stockholm Regions cohort of 1,782,125 persons followed between the 1st of March 2020 to the 28th of February 2021

	Hospital Admissions	Mortality
	RR^c^ (95% CI)	RR^c^ (95% CI)
**Sex**		
Women	1.0	1.0
Men	1.60 (1.53–1.66)	2.50 (2.19–2.85)
**Age groups**		
18–44 years	1.0	1.0
45–59 years	2.55 (2.40–2.70)	5.97 (3.97–8.99)
60–74 years	3.47 (3.25–3.70)	17.08 (11.54–25.28)
75–110 years	4.95 (4.57–5.36)	53.78 (36.00–80.33)
**Region of origin (birth)**		
Sweden	1.0	1.0
Not born in Sweden	2.11 (2.02–2.21)	1.68 (1.47–1.92)
**Living conditions**^a^		
Single household	1.0	1.0
Two person	1.05 (0.99–1.11)	0.85 (0.74–0.98)
Three person	1.25 (1.17–1.33)	1.08 (0.86–1.34)
Four person	1.30 (1.21–1.40)	0.84 (0.62–1.14)
>Four person	1.46 (1.36–1.57)	1.03 (0.79–1.35)
**Education**		
High	1.0	1.0
Medium	1.15 (1.10–1.21)	1.16 (0.99–1.36)
Low	1.38 (1.31–1.50)	1.49 (1.25–1.77)
Other	0.89 (0.84–0.94)	1.35 (1.08–1.69)
**Type of work**^b^		
Full-time	1.0	1.0
Part-time	0.77 (0.72–0.83)	0.94 (0.69–1.29)
Health worker	1.45 (1.34–1.58)	1.28 (0.83–1.97)
Other^b^	0.89 (0.84–0.94)	1.35 (1.08–1.69)
**Income**		
Income Q5 (highest income)	1.0	1.0
Income Q4	1.01 (0.94–1.09)	1.10 (0.84–1.42)
Income Q3	1.09 (1.02–1.18)	1.35 (1.06–1.72)
Income Q2	1.20 (1.11–1.28)	1.75 (1.40–2.20)
Income Q1	1.34 (1.24–1.44)	2.19 (1.73–2.79)
No info income	1.10 (0.92–1.30)	0.98 (0.42–2.28)
**Comorbidities**		
No	1.0	1.0
Yes	2.99 (2.86–3.13)	4.38 (3.75–5.12)

^a^Living conditions are measured as the size of the household (i.e., the number of people in the household)

^b^Possibility to work from home full-time or part-time. Other includes full-time students. Unemployed, long-term sick leave, or retired

^c^Odds ratios (here presented as relative risks) derived from a logistic regression model; fully adjusted models included all variables presented in the table

Table 3 shows the *contextual effect estimates* on hospitalization and death. This model takes all the individual effect estimates — presented in Tables 1 and 2 — into account. The geographical area factors standing out include a high percentage of people not born in Sweden; mortality in such areas was elevated by over 50% compared to reference areas. We also found high population density and the age distribution in an area to be associated with increased risk of both hospital admission and mortality, where a high proportion of younger adults and a low proportion of older adults were associated with increased risks.

**Table 3:** Frequency table of contextual effect estimates and variables used in model based on tertiles of percentage distributions of selected factors over geographical areas in Stockholm county

	Number of hospital admissions (%)	Number of deaths (%)
**Categories (tertiles) based on the percentage of Swedish born in a geographical area**		
High % born in Sweden	2295 (22%)	261 (23%)
Medium % born in Sweden	3368 (32%)	370 (32%)
Low % born in Sweden	4832 (46%)	517 (45%)
**Categories (tertiles) based on population density in a geographical area**		
High population density	4210 (40%)	472 (41%)
Medium population density	3848 (37%)	375 (33%)
Low population density	2437 (23%)	301 (26%)
**Categories (tertiles) based on the percentage of children**^a^ **in a geographical area**		
High % children	3145 (30%)	338 (29%)
Medium% children	4504 (43%)	460 (40%)
Low % children	2846 (27%)	350 (30%)
**Categories (tertiles) based on the percentage of older adults**^b^ **in a geographical area**		
High % elderly	2534 (24%)	324 (28%)
Medium % elderly	3971 (38%)	455 (40%)
Low % elderly	3990 (38%)	369 (32%)
**Index variable based on area covariate above (tertiles)**		
High	2922 (28%)	339 (30%)
Medium	3891 (37%)	408 (36%)
Low	3682 (35%)	401 (35%)

^a^Children = 0–15 years of age

^b^Elderly = 70 years or older

Tables 4 and 5 demonstrate the interface between area factors and individual effect estimates on the risk of hospital admission and death in a stepwise fashion. This model takes all the individual effect estimates — presented in Tables 1 and 2 — into account. Hospitalization is higher in areas with many children, fewer elderly people, high density, high area deprivation, and in areas with low percentages of Swedish-born inhabitants. Mortality is to a larger extent impacted by individual risk factors (Table 5) as demonstrated by non-significant relative risks. Important exceptions include areas with high density and low percentages of Swedish-born inhabitants. Neighborhood deprivation is explained in the methods section and the first principal component explained 42.22% of the total variability and it can be interpreted directly from Additional file1: Figure S1.

**Table 4:**
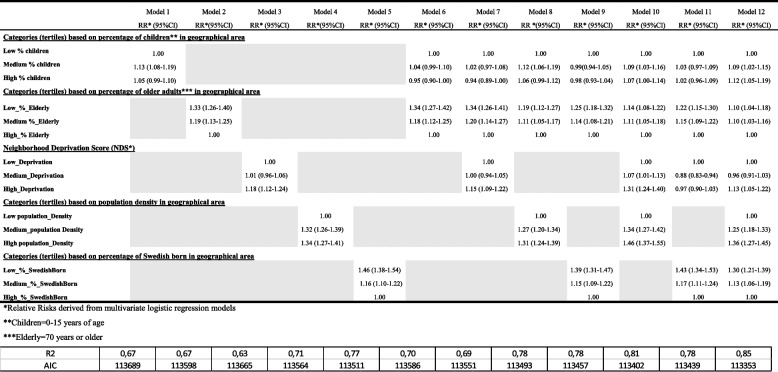
Relative risk^*^ and 95% confidence intervals of COVID-19-related hospitalization for area-level covariates (based on tertiles of percentage distributions) and a principal component analysis to generate a composite neighborhood deprivation score (NDS). Area-level covariate was included one at a time (model 1 to 12, always including the whole set of individual covariates in the models)

**Table 5:**
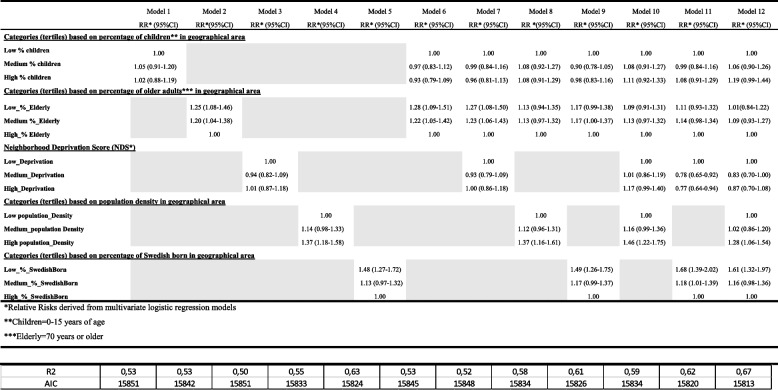
Relative risk^*^ and 95% confidence intervals of COVID-19-related deaths for area-level covariates (based on tertiles of percentage distributions) and a principal component analysis to generate a composite neighborhood deprivation score (NDS). Area-level covariate was included one at a time (model 1 to 12, always including the whole set of individual covariates in the models)

Additional file 1: Table S1 shows the crude risk of death depending on where you live and the risk after multiple adjustments. As mentioned in the methods, residential areas were divided into quintiles with the 20% most afflicted, i.e., the highest number of deaths, in quintile 1 and the 20% least afflicted in quintile 5. Crude data indicate a quadrupled death toll in the worst-off areas. Even after adjustments for sex, age, country of origin, education, type of work, income, living conditions, and co-morbid conditions; mortality is twice as high for people living in the most afflicted neighborhoods.

## Discussion

To our knowledge, this large, population-based study is the first to assess the combination of individual- and area-level risk factors on outcomes of COVID-19 patients. Our key finding is that segregation and deprivation are strongly associated to detrimental outcomes of COVID-19.

Individual factors like lower income, lower education, and inability to work from home together with age, male sex, and co-morbid conditions increase the risk of hospitalization and death. Country of origin plays a part in the individual risk of severe outcomes, but we also demonstrate group-level effects. Residence in an area with a higher percentage of immigrants was independently associated with an increased risk of hospital admission. Living in affluent neighborhoods, with few immigrants, was associated with both lower risk of hospitalization and death. There were other signals in the contextual effect estimates; areas with low numbers of children and a high percentage of people over 70 years were associated with decreased risk of hospital admission. Living in the most deprived areas in Stockholm was an independent risk factor for hospitalization during the first year of the COVID-19 pandemic.

One ecological study from Germany, during the first wave, showed COVID-19 cases and death negatively associated with the share of schoolchildren and children in day care as well as physician density [18]. In contrast to that and other ecological studies, the present investigation combines individual- and area-related factors. A Norwegian study of immigrants indicated that crowded housing and low income at a group level were correlated with cases and hospitalization of COVID-19 [19]. A nationwide Swedish study [20], albeit lacking data on hospitalization and solely examining the first wave (less than 2 months of follow-up) of the pandemic, scrutinizes sociodemographic factors. Being male, low individual income, lower education, and not being married independently predict a higher risk of death from COVID-19 and from all other causes of death. Immigrants from low- or middle-income countries had a higher risk of COVID-19 mortality but not for other causes of death.

The Stockholm data presented here augments this base of knowledge. On top of adding very granular individual and group-level information, we include data on hospitalization with over two years of follow-up encompassing five distinct COVID-19 waves. The greater Stockholm area has been the epicenter of the pandemic in Sweden and indeed in all of Scandinavia [21]. This could partly be explained by the fact that the region shares many traits of other European large cities with regard to precarious employment, crowded housing, and foreign-born inhabitants. The fact that individual factors, such as older age, lower income, less education *as well as contextual factors* — the type of area one resides in — are all associated with increased risk of hospitalization or death is paramount for politicians, policy makers, and health care personnel. This knowledge allows for targeted outreach, when striving to contain potential new variants of the SARS-CoV-2 virus, or indeed other transmissible diseases. To be specific, exploring and explaining the intersection between risk factors at the geographical area level and individual risk factors has implications for our ability to intervene in an optimal way. If, for example, the individual age was the most important factor, interventions need to reach that demographic group independently of where they live. However, as clearly demonstrated here, certain geographic area traits carry *independent additional risk*; interventions need to be focused towards high-risk areas and especially on old people residing in such areas. Support focused on those not born in Sweden could have had significant health benefits during the first year of the pandemic and must be improved in the future.

This investigation has strengths. The Region Stockholm real-time COVID-19 monitoring framework makes a detailed analysis of risk factors for COVID-19-related mortality and hospitalization quite simple. We have complete coverage of the entire greater Stockholm area population and all deaths and hospitalizations due to COVID-19 for the whole study period. Data resolution is exceptional, with individual data on education, income, work situation, living area, and living condition as well as the country of origin and co-morbid conditions. Additionally, we present unique sociodemographic properties on an area level; including population density, average income, age groups and data on foreign-born inhabitants. *The Stockholm Syndrome* described here, with large swaths of low-income workers, unable to work from home, and residing in crowded households may, as mentioned above, be quite generalizable. These are conditions rampant in many larger cities across the continent and in the world. These findings and these circumstances are likely to have an impact beyond COVID-19. Yet, the findings should be interpreted with caution. Swedish data on COVID-19 deaths is considered accurate, the National Board of Health and Welfare reports all cases where the underlying cause of death was COVID-19, regardless of whether the diagnosis was laboratory-confirmed or not [22]. Persons ill enough to seek hospital care will be admitted (the Swedish health care system is not based on private insurance). However, it is impossible to rule out that inequalities in hospitalization due to COVID-19 might partly be affected by differences in health-seeking behavior. Certain individuals may have a lower bar for seeking medical attention. In contrast, others may have the ability to “navigate” the health care system more efficiently; one needs to be aware of this when using hospital admission as a proxy for the severity of the disease. With this said, it is not self-evident in which direction this would skew the risk estimates. Additionally, some findings, like the fact that areas with more elderly people had a lower risk of hospitalization may seem counterintuitive. We can only speculate, but the adherence to regional and national recommendations, such as avoiding shopping, avoiding crowds, and general stay-at-home-recommendations, is likely higher among the elderly. Dichotomizing Stockholm residents into born in Sweden vs not born in Sweden and not taking into account time spent in Sweden may blunt our ability to draw conclusions regarding this variable. Lastly, the risks of death in areas that were hit hard by the pandemic could — despite our best efforts to adjust for all individual factors — be driven by residual confounding. At the same time — for policy makers in the healthcare sector — that does not matter. Our findings clearly indicate where and to whom we should focus governmental resources.

## Conclusions

segregation and deprivation are well-known public health issues. This study from Stockholm, Sweden shows that the risk of hospitalization and death from COVID-19 is increased by area factors, such as population density, the number of people born in Sweden on top of aspects like the inability to work from home and living in crowded housing. In combination, these add to known individual risk factors such as male sex, high age, and cardiac, renal, liver, and other comorbidities [15]. Even when correcting for all known individual risk factors, residing in the highest-risk areas is associated with a more than doubled risk of dying from COVID-19. This finding, in conjunction with the aforementioned individual risk factors, is paramount for governments, agencies, and healthcare institutions interested in targeted interventions such as vaccine outreach for the elderly residing in high-risk areas. The interaction between sociodemographic factors and the virus is very clear; an insight that can help how we deal with future strains of SARS-CoV-2 or other pandemics.

## Supplementary Information

**Additional file 1: Figure S1.** Area level covariates for the principal components analysis, creating the neighborhood deprivation score. **Table S1.** Risk of covid-19 death among residents 18 years and older (*n*= 1 722 444) divided by residential area* in Stockholm Region. Follow-up between 1^st^ of March 2020 to the 28^th^ of February 2021, Individuals living in elderly care facilities or with home-care service excluded.

## Data Availability

Swedish privacy law prohibits us from making registered data publicly available. The data supporting our findings were used under license and ethical approval for the current study. Readers interested in obtaining microdata or replicating our study may seek similar approvals and inquire through Statistics Sweden. For further advice see: https://www.scb.se/en/services/guidance-for-researchers-and-universities/, or contact Statistics Sweden at: mikrodata@scb.se.

## Abbreviations

CI: Confidence interval
COVID-19: The coronavirus disease 2019
ISCO: International standard classification of occupations
LISA: Integrated Database For Labor Market Research
NDS: Neighborhood deprivation score
RR: Relative risk
RTB: Total Population Register
SARS-CoV-2: Severe acute respiratory syndrome coronavirus 2
SSYK: Standard for Swedish occupational classifications
VAL: Swedish: Vård Analys Lager, the Stockholm Regional Healthcare Data Warehouse
