# Supplementary material for: Individual and neighborhood risk factors of hospital admission and death during the COVID-19 pandemic: a population-based cohort study
Source: BMC Med. 2023 Jan 4;21:1. doi: 10.1186/s12916-022-02715-4 (PMC9812348; doi:10.1186/s12916-022-02715-4)
Supplement: Supplementary file 1 — Additional file 1: Figure S1. Area level covariates for the principal components analysis, creating the neighborhood deprivation score. Table S1. Risk of covid-19 death among residents 18 years and older (n= 1 722 444) divided by residential area* in Stockholm Region. Follow-up between 1st of March 2020 to the 28th of February 2021, Individuals living in elderly care facilities or with home-care service excluded. [file 12916_2022_2715_MOESM1_ESM.docx]

**ADDITIONAL FILE 1 TABLES AND FIGURES**

**Additional figure 1: Area level covariates for the principal components analysis, creating the neighborhood deprivation score
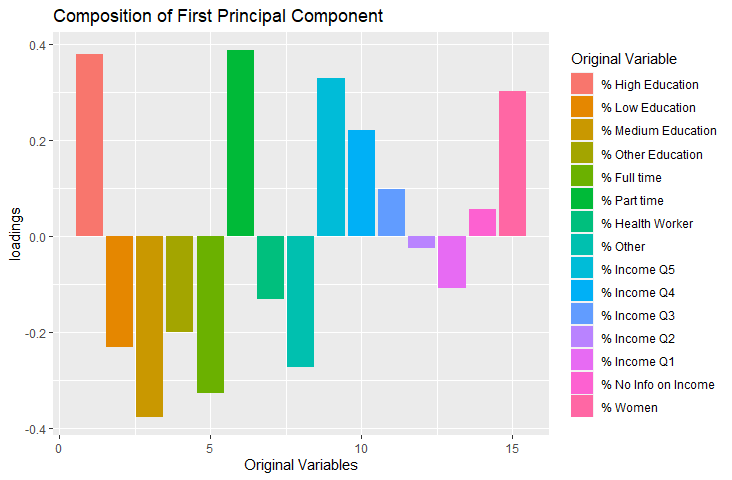
**

| **Additional table 1: Risk of covid-19 death among residents 18 years and older (n= 1 722 444) divided by residential area* in Stockholm Region. Follow-up between 1^st^ of March 2020 to the 28^th^ of February 2021, Individuals living in elderly care facilities or with home-care service excluded** | | | | | | |
| --- | --- | --- | --- | --- | --- | --- |
|  |  |  | Model 1 | Model 2 | Model 3 | Model 4 |
|  | Number of deaths | Personyears | HR (95% CI) | HR (95% CI) | HR (95%) | HR (95%) |
| Quintile 1** | 363 | 320 663 | 2.61 (2.08-3.29) | 2.52 (2.00-3.18) | 2.45 (1.95-3.10) | 2.44 (1.94-3.08) |
| Quintile 2 | 242 | 325 483 | 2.19 (1.73-2.77) | 2.17 (1.71-2.75) | 2.14 (1.69-2.71) | 2.10 (1.65-2.66) |
| Quintile 3 | 215 | 374 540 | 1.77 (1.39-2.26) | 1.76 (1.39-2.25) | 1.76 (1.38-2.24) | 1.77 (1.39-2.25) |
| Quintile 4 | 161 | 364 456 | 1.26 (0.97-1.62) | 1.26 (0.98-1.63) | 1.27 (0.98-1.64) | 1.28 (0.99-1.65) |
| Quintile 5 (ref) | 90 | 313 490 | 1 | 1 | 1 | 1 |
| *Residential area is divided into quintiles with the 20 percent most affected (death cases) in group 1 and the 20 percent least affected in group 5.  **Where less affected in the second and third wave compared to the first wave.  Model 1: adjusted for sex, age and country of birth  Model 2: adjusted for sex, age, country of birth and education (post-secondary, secondary, pre-secondary and other)  Model 3: adjusted for sex, age, country of birth, education (post-secondary, secondary, pre-secondary and other), type of work and income (quintiles)  Model 4: adjusted for sex, age, country of birth, education (post-secondary, secondary, pre-secondary and other), type of work, income (quintiles), living condition (pop density per square meter) and comorbidity | | | | | | |
